# Incorporation of docetaxel and thymoquinone in borage nanoemulsion potentiates their antineoplastic activity in breast cancer cells

**DOI:** 10.1038/s41598-020-75017-5

**Published:** 2020-10-22

**Authors:** Mayson H. Alkhatib, Raghdah S. Bawadud, Hana M. Gashlan

**Affiliations:** Department of Biochemistry, Faculty of Science, King Abdulaziz University, Jeddah, Saudi Arabia

**Keywords:** Biophysics, Cancer, Drug discovery, Medical research, Oncology, Nanoscience and technology

## Abstract

Combining more than one anticancer agent in a nanocarrier is beneficial in producing a formula with a low dose and limited adverse side effects. The current study aimed to formulate docetaxel (DTX) and thymoquinone (TQ) in borage oil-based nanoemulsion (B-NE) and evaluate its potential in impeding the growth of breast cancer cells. The formulated B-NE and the combination (DTX + TQ) B-NE were prepared by the ultra-sonication method and physically characterized by the dynamic light scattering techniques. The cytotoxicity analyses of (DTX + TQ) B-NE in MCF-7 and MDA-MB-231 cells were evaluated in vitro by using the SRB assay. Cell death mechanisms were investigated in terms of apoptosis and autophagy pathways by flow cytometry. The optimum mean droplet sizes formulated for blank B-NE and the (DTX + TQ) B-NE were 56.04 ± 4.00 nm and 235.00 ± 10.00 nm, respectively. The determined values of the half-maximal inhibitory concentration (IC_50_) of mixing one-half amounts of DTX and TQ in B-NE were 1.15 ± 0.097 µM and 0.47 ± 0.091 µM in MCF-7 and MDA-MB-231 cells, respectively, which were similar to the IC_50_ values of the full amount of free DTX in both tested cell lines. The treatment with (DTX + TQ) B-NE resulted in a synergistic effect on both tested cells. (DTX + TQ) B-NE induced apoptosis that was integrated with the stimulation of autophagy. The produced formulation enhances the DTX efficacy against human breast cancer cells by reducing its effective dose, and thus it could have the potential to minimize the associated toxicity.

## Introduction

Breast cancer is considered as invasive cancer among females and the fifth type of cancer that leads to cancer death^1^. Therefore, it is essential to gain an understanding of the complexities of the mechanism of action for the combined treatments in breast cancer cells in order to identify the optimal treatment strategy^2,3^. Taxane treatments, which is extracted mainly from the European yew tree needles, have remained a cornerstone of breast cancer treatment for the past three decades^4^. Docetaxel (DTX) is a second-generation taxane and it has a wide spectrum of antitumor activity mainly by promoting the stabilization of cellular microtubules, thereby inhibiting cell division^2,5^. However, the narrow therapeutic index of DTX restricts its anticancer activity. In addition, the developed drug resistance, poor solubility, and toxic effects of DTX are considered major limitations^4,6^. Therefore, refining taxane-based regimens by combining with novel agents has gained a rising interest in clinical research^2^.

Combination therapy involves the administration of two or more treatments with various mechanisms of action in order to overcome drug resistance and to reduce the effective dose of chemotherapy and thus the side effects. Consequently, combination therapy with a synergic effect would result in increased therapeutic efficacy^2,7^. Recently, great attention is paid to exploit bioactive components of natural plants having anticancer activity. The major bioactive component extracted from the black seed oil of the *Nigella sativa* plant is thymoquinone (TQ)^8,9^. The biological activities of TQ have gained an increasing interest mainly its anti-inflammatory and anticancer properties. Owing to the interference with multiple pathways involved in the processes of angiogenesis, invasion, and metastasis, TQ has the ability to inhibit cancer development^10^. Thus, the conjunction of TQ with chemotherapeutic agents, such as doxorubicin, cisplatin, oxaliplatin, gemcitabine, and 5-fluorouracil have been investigated to improve their activity against several types of cancer^7,11,12^.

A successful co-delivery of combined therapeutic agents can be challenging because of the potential differences among their physiochemical and pharmacodynamic properties^13^. Moreover, several combined therapies suffer from a limited penetration to the cytoplasmic area and off-targeting effects^6^. In order to accomplish a targeted synergistic cytotoxic effect, a nano-delivery platform was therefore designed to co-deliver different therapeutic agents and maximize their efficacy. Oil-in-water nanoemulsions (NEs) are defined as oil droplets dispersed in an aqueous phase, at which each droplet, surrounded by surfactant molecules, has a size typically in the range of 20–500 nm^14^. This drug delivery system has gained interest nowadays due to its unique physical properties such as the extremely small droplet diameter with the special optical transparency and elastic properties compared with other conventional emulsions^15^. The NEs are used in drug delivery research mainly because of its high potential in enhancing the bioavailability and efficiency of loaded drugs while reducing the toxic effects^16,17^. Borage flower is commonly known as the richest source of gamma-linolenic acid (GLA), an essential omega-6 fatty acid^18^. Increasing evidence suggests that GLA has been proposed as a valuable new cancer therapy having selective toxicity against cancer cells^19^. For example, in vitro studies have demonstrated that GLA improves the effectiveness of DTX against breast cancer cells^20^. Hence, borage oil has been selected in the NE synthesis due to its therapeutic potential as it would positively support the suggested co-delivery system.

In this research, a new formulation was proposed to enhance the efficacy of DTX by mixing with TQ in a NE system formulated with borage oil using a high energy method. The anticancer activities of the produced nano-formulation were examined in the MCF-7 and MDA-MB-231 cells.

## Materials and methods

### Materials

The oils of Borage and Medium Chain Triglyceride (MCT) were obtained from the electronic store iHerp.com. Tween 80 and Span 20 were purchased from Al Shafei medical and Scientific Equipment, Est (Jeddah, KSA). Docetaxel (DTX) was kindly provided by King Abdulaziz University Hospital. Thymoquinone (TQ) and Sulforhodamine B (SRB) dye were obtained from Sigma-Aldrich (USA). The Coomassie blue dye was purchased from Cayman Chemical (Michigan, US). Annexin V-FITC apoptosis detection kit was obtained from Invitrogen Abcam (UK). Acridine Orange and rapamycin were obtained from BDH Chemicals Ltd (England, UK) and Enzo Life Sciences (Lausen, Switzerland), respectively. Human Breast cancer cell lines, MCF-7 and MDA-MB-231, were obtained from ATCC (American Type Tissue Culture Collection, Manassas, VA, USA).

### Formulation of B-NE formulas

The blank oil-in-water (O/W) NE was prepared under optimal conditions for ultrasonic emulsification, typically through a two-stage process^21^. At first, it was prepared by adding 5.55 % (v/v) of the combination oils of borage oil and MCT oil at the ratio of (1:1) into 75 % (v/v) of buffer solution with pH 8. Then, 19.45 % (v/v) of pre-warmed surfactant, Tween 80, and co-surfactant, Span 20, were added at a constant ratio of (2:1), respectively. The ratio between oils to surfactants (O/S) was maintained at (1:3.5), as this formulation exhibited the optimal stability. The resulted mixture was then vortexed for approximately 5 min at room temperature, until emulsions developed as one phase of a milky liquid.

Secondly, all samples were sonicated using Omni Sonic Ruptor 4000 Ultrasonic Homogenizer (Kennesaw, GA, USA) equipped with the ultrasound probe of titanium, and 3.8 mm in diameter. Throughout sonication, the tip was symmetrically immersed 1.0 cm above the end of the tube and the heat energy generated was neutralized by keeping the sample tubes in a glass beaker with ice at 4 °C to prevent overheating. The sonication was carried out for approximately 20 min until a clear and transparent phase formed indicating the production of B-NE. Each experiment was performed in triplicate (n = 3).

In general, the (DTX + TQ) B-NE samples were prepared by mixing (1:1) of docetaxel (DTX) and thymoquinone (TQ) followed by adding it directly into freshly prepared blank B-NE to produce a stock concentration of 100 µM. It was then further diluted to the desired intermediate concentrations depending on each experiment.

### Physical characterizations of B-NE formulas

#### Droplet size analysis

The average droplet sizes (z-average diameter), polydispersity index (PDI), and surface charge (zeta potential) values of all B-NE samples were identified by dynamic light scattering (DLS) technique (Malvern Zetasizer Nano ZS instrument, UK) equipped with Malvern Zetasizer software (version, 6.32). Typically, the analyses were performed with a light scattering angle of 173° and at 25 °C. All samples were measured in triplicates. The collected data were analyzed as intensity-and volume-weighted particle size distribution.

#### Stability

The stability constant ${(\text{K}}_{\text{S}}$) was used to assess the stability of blank B-NE and (DTX + TQ) B-NE. It was determined by the centrifugation-spectrophotometric method as previously described with modifications^22,23^. The original absorbance of B-NE samples (${\text{A}}_{0}$) was first measured by using GENESYS 10S Vis Spectrophotometer at 272 nm. Then, the centrifugation of 1.0 mL for each sample was performed at 3000 rpm for 10 min, followed by removing the supernatants. To obtain the sample absorbance (A), the bottom sample was then measured. The ${\text{K}}_{\text{S}}$ value was then calculated as the following equation: ${\text{K}}_{\text{S}}$ = (${\text{A}}_{0}$ – A) /${\text{A}}_{0}$. It should be noted that a smaller ${\text{K}}_{\text{S}}$ value indicates little precipitating or floating of NE formula, which implies good stability.

### Drug release (in vitro)

The release profiles of free (DTX + TQ) and the (DTX + TQ) B-NE were evaluated in vitro by the dispersion technique using the dialysis bag procedure^24^. To simulate the blood circulation environment, the experiments were conducted in phosphate-buffered saline (PBS, pH = 7.4). Briefly, (2 mL) of each sample was loaded into a dialysis bag (Mw cut-off = 3.5 kDa) and immersed in 100 mL release media having 1 % Tween 80 in PBS as a solubilizer to achieve sink conditions. Samples were then maintained at a constant shaking mixer at room temperature. Aliquots (1 mL) were collected at appropriate time intervals up to 24 h and replaced with fresh media to maintain a constant volume. Finally, the collected samples were measured at 272 nm using GENESYS 10S Vis Spectrophotometer (Thermo Fisher Scientific, USA). For analysis, the cumulative releases of drugs in percentages were plotted vs. time to assess the release profiles. All measurements were carried out in triplicate.

### Cell cultures

The MCF-7 and MDA-MB-231 cells were cultured in (DMEM) media supplemented with 10 % (v/v) fetal bovine serum (FBS) and 1 % (v/v) penicillin–streptomycin (Gibco Invitrogen, Carlsbad, CA, USA), followed by incubation in a humidified 5 % CO_2_ atmosphere at 37 °C. Upon reaching 90 % confluence, cells were routinely sub-cultured with a split ratio of 1:4 by trypsinization (0.25 % Gibco-trypsin; Gibco, Thermo Fisher Scientific, KSA) to maintain optimum growth and viability of cultured cells.

### Cytotoxicity assay

The sulforhodamine B (SRB) assay was used to verify the cytotoxicity of DTX, TQ, and (DTX + TQ) B-NE in MCF-7 and MDA-MB-213 cells as previously described^20,25^. In brief, cultured cells were seeded in 96-well plates at a seeding density of 1 $\times \; {10}^{4}$ cells/mL. On the following day, cells were treated for 48 h with the serial concentrations of DTX, TQ, B-NE, and free (DTX + TQ) or the loaded (DTX + TQ) B-NE, ranged from (0.01 to 100 µM). The combined drugs DTX and TQ were mixed in the ratio of (1:1) in either distilled water or B-NE to prepare a stock solution of 100 µM of free (DTX + TQ) or (DTX + TQ) B-NE, respectively. Afterward, the fixation of treated cells with trichloroacetic acid (TCA) (10 % w/v) was performed for one hour at 4 °C. Fixed cells were washed with distilled water and exposed to SRB dye (0.4% (w/v)) for 10 min at room temperature in the dark and subsequently washed by using 1 % (v/v) acetic acid to carefully remove all excess dye. Plates were then allowed to dry overnight. Finally, the SRB-stained cells were dissolved in Tris–HCl (50 mM, pH 7.4) and the product of the reaction was measured at 540 nm with a microplate reader (BioTek, USA). Cell viability was expressed as a percentage of values calculated from three replicates of each tested treatment, as shown in the following equation:

$$\text{\%Cell} \;\text{viability}=\left(\frac{Abs\; of \;treated \;cells-Abs \;of\;blank}{Abs\; of\; control-Abs \;of \;blank} \times 100\right)$$

All treatments of the dose–response curves and their half-maximal inhibitory concentration (IC_50_) values, which is the drug concentration required for 50 % inhibition of cell proliferation, were analyzed using Prism (version 8.0, GraphPad Software Inc., La Jolla, CA, USA). In addition, the combination index (CI) values were used to evaluate the nature of drugs interaction which are identified as antagonism if CI > 1.3; additive if CI (0.9–1.1); slight synergism if CI (0.8 – 0.9) and synergism if CI (0.4–0.8)^7,26,27^. The CI values were calculated from the formula:

$$\text{CI}=\frac{{\text{IC}}_{50 }\;\text{drug }\left(\text{ a}+\text{b }\right)\text{combination}}{{\text{IC}}_{50}\;\text{ drug }\left(\text{ a }\right)\text{alone}}+\frac{{\text{IC}}_{50}\;\text{ drug }\left(\text{ a}+\text{b }\right)\text{combination}}{{\text{IC}}_{50}\;\text{ drug }\left(\text{ b }\right)\text{alone}}$$

### Characterization of cell morphology

The morphologies of treated cells with the free DTX, TQ, and B-NE or (DTX + TQ) B-NE were investigated using the light microscope as previously reported with modifications^28^. In general, cultured cells were seeded into 96-well, flat-bottomed plates. On the following day, cells were incubated with 200 μL of media containing the pre-determined IC_50_ of each treatment for 48 h at 37 °C and 5 % CO_2_ incubator. Next, 100 μL of PBS (pH 7.2) was used to wash cells twice. Cells were fixed by the addition of 4 % formaldehyde for 15 min. The cells staining was then performed by adding 10 % Coomassie blue dye for 10 min. Lastly, the stained cells were washed twice with distilled water and allowed to dry overnight. The morphology of the cells upon treatment was then observed with an inverted microscope at 40× magnifications (TH4-200, Olympus optical Co-Ltd, Tokyo, Japan).

### Evaluation of the apoptosis

The mechanism of cell death analysis in response to treatments was performed with the Annexin V-FITC assay (Abcam, Cambridge, UK) according to the manufacturer’s instructions. Briefly, cells grown in 6-well plates at a density of 1 × ${10}^{5}$ cells/well were exposed to the predetermined IC_50_ values of each treatment for 48 h. Then, cells were harvested, washed with PBS, and incubated with 0.5 mL of Annexin V-FITC/PI (propidium iodide) solution for 30 min in the dark at room temperature. Samples were measured by flow cytometry (BD FACS Aria III). The flow cytometry plots were analyzed using FACS Diva Version 6.1.3 software.

### Autophagy assay

The acidic vesicular organelles (AVOs), a morphological characteristic of autophagy, was identified with the acridine orange staining (Sigma-Aldrich, St. Louis, MO) as previously described with modifications^29,30^. In general, grown cells in 6-well plates at a density of 1 × ${10}^{5}$ were treated with the pre-determined IC_50_’s of the tested formulas for 48 h. The positive control cells were exposed to (10 μg/well) of rapamycin which is an autophagy inducing agent. Following treatment, collected cells were washed with PBS and then stained for autophagic vacuoles using 1 μg/mL acridine orange for 30 min in the dark at room temperature. Fluorescent autophagic vacuoles were analyzed via Flow Cytometer (BD FACS Aria III) and quantified using the FACS Diva Version 6.1.3 software.

### Statistical analysis

All measurements were performed at least in triplicate and were reported as the mean ± standard deviation (SD). Data were analyzed by one-factor analysis of variance (ANOVA) using Prism, version 8.00, for Mac OS X (GraphPad Software, CA, USA). Differences among samples were considered statistically significant at p-value < 0.05.

## Results

### Physical characterization of B-NE formulas

The size, charge, and physical state of the formulated NEs play a key role in the functional performance as a delivery system^31^. As displayed in Table 1, the z-average diameters for both of blank B-NE and (DTX + TQ) B-NE were within the desired size range with a narrow size distribution. Curiously, the nanodroplet sizes of the (DTX + TQ) B-NE were markedly larger than blank B-NE, suggesting the successful entrapment of loaded drugs. In addition, the zeta potential values of the (DTX + TQ) B-NE have become smaller than that of the blank B-NE. In spite of the differences in the stability constants (*K*s) for blank B-NE and (DTX + TQ) B-NE, their minimal amounts for both of them suggest suitable stability.

**Table 1:** Physical characteristics of the B-NE formulations.

Formulation code	Z-average diameter (nm)	Zeta potential (mV)	Polydispersity index (PDI)	Stability constant (Ks)%
Blank B-NE	56.04 ± 4.00***	− 0.91 ± 0.20***	0.071 ± 0.020	10.90 ± 0.02***
(DTX + TQ) B-NE	235.00 ± 10.00	− 0.38 ± 0.100	0.042 ± 0.020	14.01 ± 0.03

The data were expressed as the mean ± SD (*n* = 3). ***P < 0.001, the blank B-NE vs. the corresponded (DTX + TQ) B-NE, (one-factor ANOVA).

### Drug release (in vitro)

**Figure 1:**
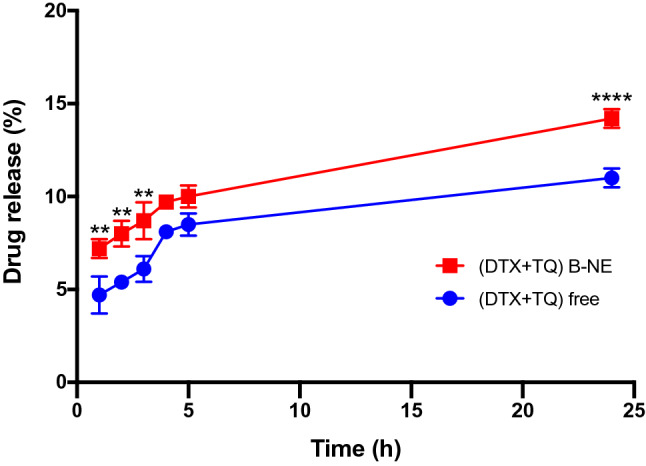
In vitro drug release of the combination treatments. Comparison between in vitro drug release profiles of the (DTX + TQ) free and (DTX + TQ) B-NE as a function of time in PBS (0.01 M, pH = 7.4 containing 0.1 % (v/v) of Tween 80). All values were expressed as mean ± SD (n = 3). **P < 0.0021, ****P < 0.0001 in comparison to (DTX + TQ) free, assessed by one-factor ANOVA.

The free drugs combination (DTX + TQ) and (DTX + TQ) B-NE revealed initial stage release followed by sustained release of drugs within 24 h. The release of (DTX + TQ) B-NE was considerably higher when compared to the free drugs release profile as shown in Fig. 1.

### Cytotoxic activity

**Figure 2:**
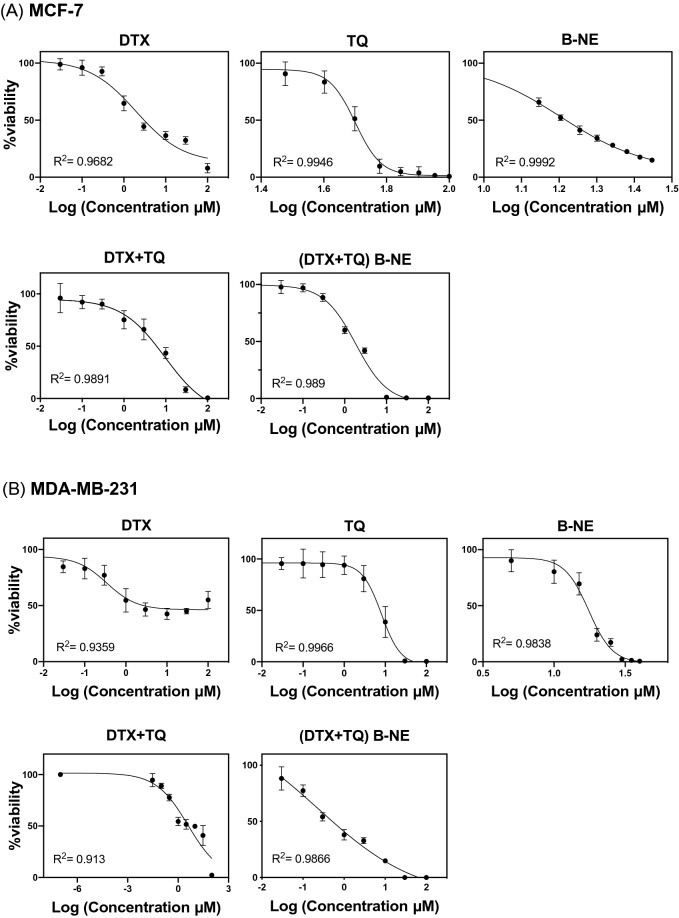
The dose–response curves of DTX, TQ, B-NE, DTX + TQ-free and the loaded (DTX + TQ) B-NE. (**A**) MCF-7 and (**B**) MDA-MB-231 cells were exposed to serial dilution of the tested drugs for 48 h. The cell viability was determined by the SRB assay. Data were represented as mean ± SD (n = 3).

The findings of SRB assay revealed that the trend line of the % cell viability is decreasing at a steady rate in both tested cell lines when exposed to the tested formula indicating a dose-dependent cytotoxicity effect (Fig. 2). The discrepancies in the shapes of the curves imply different mechanisms of actions of DTX, TQ, and B-NE. When the tested formulas were applied to the MCF-7 and MDA-MB-231 cells, the determined IC_50_ values were the least for both (DTX + TQ) B-NE and free DTX (P < 0.05) implying that they have the best inhibition effect (Table 2). In spite of the minimal cytotoxic effect of the single treatments, TQ and B-NE, on MCF-7 and MDA-MB-231 cells, mixing one-half amounts of DTX and TQ in B-NE gave similar cytotoxic effect to the full amount of free DTX but with a different mechanism of action as presented in the shapes of the curves in Fig. 2(A,B). According to the calculated CI values displayed in Table 2, the drugs in (DTX + TQ) B-NE have synergistic and slight synergistic effects on the MCF-7 and MDA-MB-231 cells, respectively. In contrast, combining DTX with TQ in distilled water resulted in antagonistic effects on both tested cell lines.

**Table 2:** Summary of cytotoxic parameters of DTX, TQ, B-NE, and their combinations against breast cancer cell lines, MCF-7 and MDA-MB-231, after 48 h treatment.

Treatments	IC_50_ (µM)	
	MCF-7	MDA-MB-231
DTX	1.94 ± 0.55	0.55 ± 0.22
TQ	24.0 ± 5.0****	8.07 ± 2.50***
B-NE	16.0 ± 2.0***	17.0 ± 4.00****
DTX + TQ	7.56 ± 1.00 CI-value = 4.21 Antagonism***	1.08 ± 0.58 CI-value = 2.09 *Antagonism*
(DTX + TQ) B-NE	1.15 ± 0.097 CI-value = 0.6 *Synergism*	0.47 ± 0.091 CI-value = 0.9 *Slight synergism*

(IC_50_) values denote the half maximal inhibitory concentration. (CI) values indicate the combination index as antagonism (CI > 1.3), slight synergism (0.8–0.9) and synergism (CI < 0.8). The data were expressed as mean ± SD (n = 3). ***P < 0.001 in comparison to DTX (one-factor ANOVA).

### Morphological assessment of MCF-7 and MDA-MB-231 cells

**Figure 3:**
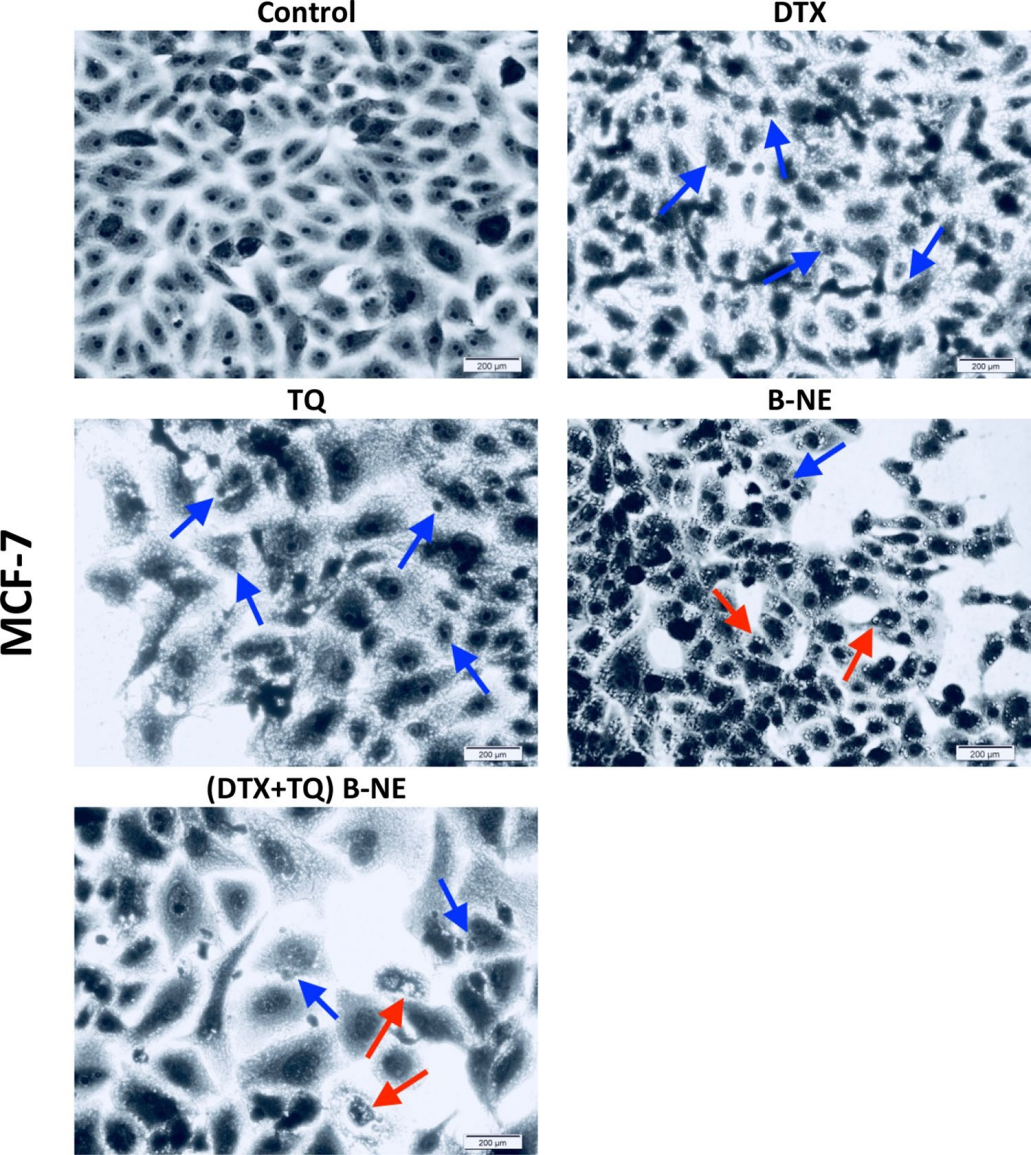
Morphological changes induced in MCF-7 cells. At culturing conditions, cells were treated with the pre-determined IC_50_’s of DTX, TQ, B-NE either as single or their combination for 48 h. Cells were stained with Coomassie Blue dye and photos were then taken using light microscopy. (Scale bar = 200 µm). Blue arrows represent shrinkage of cells, nuclear fragments, and apoptotic bodies while red arrows represent cytoplasmic blebs.

**Figure 4:**
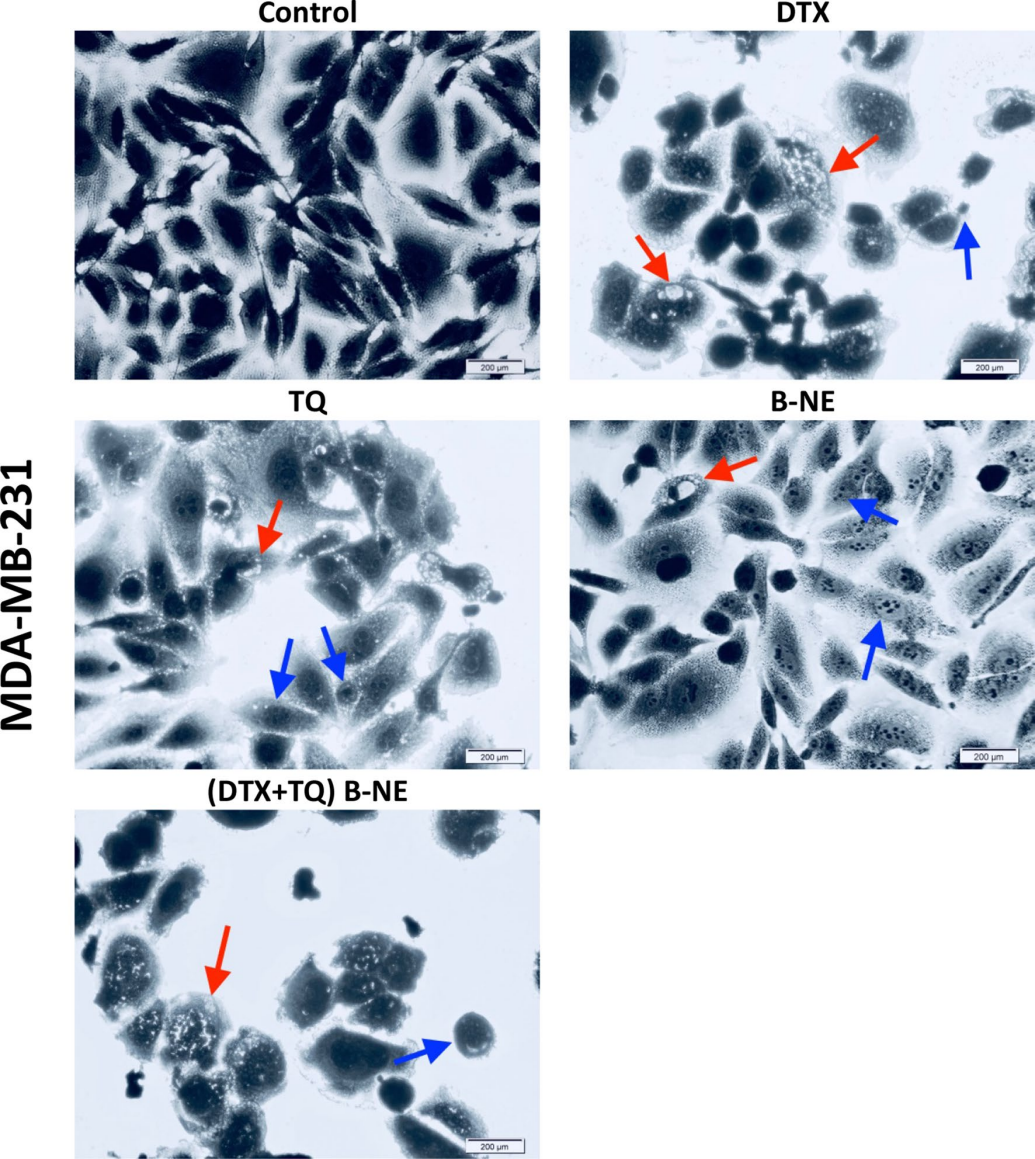
Morphological changes induced in MDA-MB-231 cells. At culturing conditions, cells were treated with the pre-determined IC50’s of DTX, TQ, B-NE either as single or their combination for 48 h. Cells were stained with Coomassie Blue dye and photos were then taken using light microscopy (Scale bar = 200 µm). Blue arrows represent shrinkage of cells, nuclear fragments, and apoptotic bodies while red arrows represent cytoplasmic blebs.

The cellular morphological changes caused by treatments were observed under the light microscope after Coomassie blue staining to investigate the cell death mechanism. The MCF-7 cells treated with the free DTX and TQ have undergone damaging in the nuclei with the presence of chromatin condensation and fragmentation. In contrast, cells treated with free B-NE have endured more membrane blebbing and cell shrinkage. The (DTX + TQ) B-NE treated cells have exhibited similar alterations in morphology as the free DTX with more content leakage from the cells (Fig. 3). In the case of MDA-MB-231cells, untreated cells displayed no evidence of fragmentation in the nuclei with a more homogenous color, while cells treated with either free DTX or TQ have exhibited noticeable changes in the nuclei morphology with more cell membrane shrinkages (Fig. 4). The treated cells with free B-NE have suffered more damage in the nuclei and chromatin fragmentation. The aberrant death of cells treated with the (DTX + TQ) B-NE formula was characterized by cytoplasmic vacuolization with increased intracellular spaces and clearance of cells. Cells also revealed nuclear destruction similar to the free DTX effects.

### Assessment of apoptotic induction

**Figure 5:**
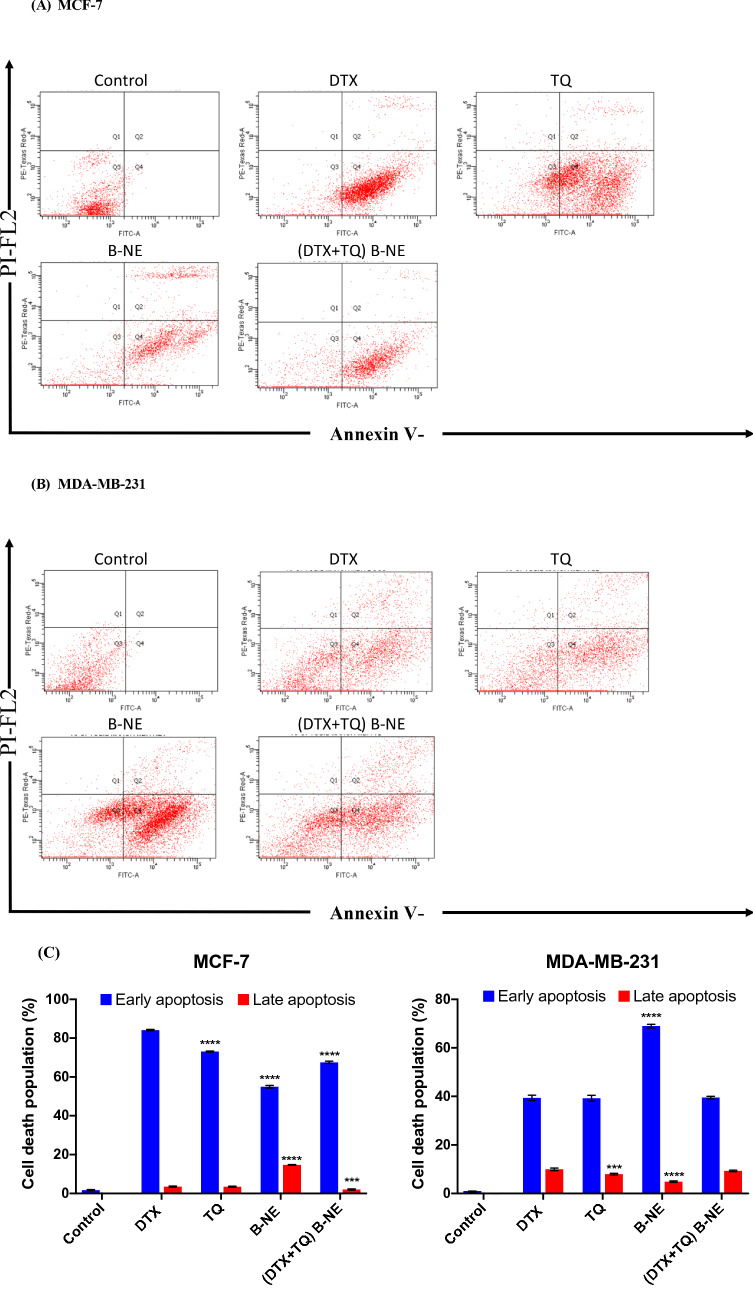
The effects of DTX, TQ, B-NE, and their combinations on apoptosis in MCF-7 and MDA-MB-231 at 48 h. Cells were treated with the pre-determined IC_50_’s of test drugs or drug-free media for 48 h. The percentage of apoptotic cells was measured using the Annexin V-FITC flow cytometry in (**A**) MCF-7 and (**B**) MDA-MB-231 cells. (**C**) A summary of the percentage of early apoptosis and late apoptosis are shown in MCF-7 and MDA-MB-231 cells. Data were presented as mean ± SD (n = 3). ***P < 0.0002, ****P < 0.0001 in comparison to DTX, assessed using one-factor ANOVA.

To investigate whether the observed growth inhibition upon DTX, TQ, B-NE, and (DTX + TQ) B-NE treatment was associated with the induction of apoptosis in MCF-7 and MDA-MB-231, cells exposed to the pre-determined IC_50_ of all tested treatments were evaluated by Annexin- V/FITC apoptosis detection assay. As exhibited in Fig. 5(A,C), the maximal increase in the percentage of MCF-7 cells undergoing early (Annexin V + /PI-) apoptosis was detected with the exposure of DTX alone (84.13 ± 0.13 %) when compared to the untreated control cells. In addition, the rates of early apoptotic cells caused by TQ, B-NE, and (DTX + TQ) B-NE treatments were 73.10 ± 0.26 %, 54.97 ± 0.65 %, and 67.53 ± 0.60 %, respectively. Although B-NE treatment had the lowest early apoptotic rates, it induced a significant level of late apoptosis at 14.17 ± 0.21 %, which was significantly better than DTX alone (3.50 ± 0.3 %, P < 0.05).

In MDA-MB-231 cells, all treated groups induced apoptosis which were significantly different from control as shown in Fig. 5(B,C). The DTX and TQ treatments induced a similar level of early apoptosis of 39.37 ± 1.10 % and 39.23 ± 1.21 %, respectively. In comparison to DTX alone, B-NE treatment resulted in a significant increase in the early apoptotic cells with 68.97 ± 0.76 % (P < 0.05). In addition, late apoptotic cells were also detected with B-NE alone treatment at 4.83 ± 0.29 %. Interestingly, when the combined DTX and TQ drugs were loaded within B-NE, a decrease in the early apoptosis was detected of 39.50 ± 0.56 %, alongside an increase in the late apoptosis induction of 9.27 ± 0.31 % when compared to the blank B-NE. Importantly, despite the lower dosage of DTX used in (DTX + TQ) B-NE compared to the DTX alone, the apoptosis analysis suggests that the combined drugs loaded B-NE performed similarly to the DTX alone. Thus, indicating an enhancement of DTX activity which is in agreement with the previous finding in the cytotoxicity analyses.

### Evaluation of the autophagy

**Figure 6:**
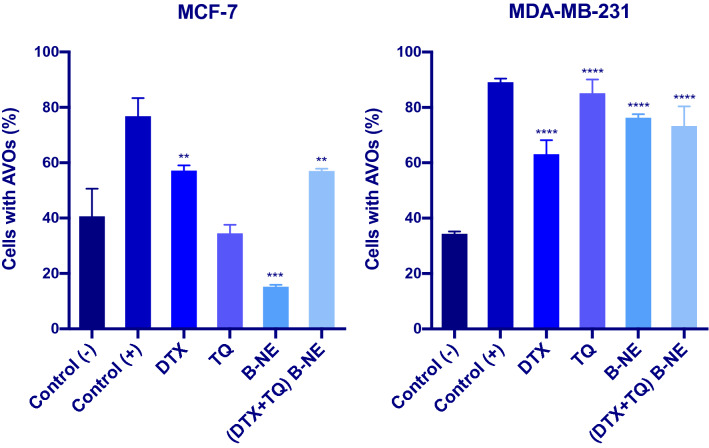
The effects of DTX, TQ, B-NE, and their combinations on autophagy in MCF-7and MDA-MB-231 Cells at 48 h. Cells were treated with the pre-determined IC_50_’s of treatments, and with rapamycin as a positive control for 48 h. Formation of acidic vesicular organelles (AVOs), a morphological characteristic of autophagy, was detected by acridine orange staining and quantified by flow cytometry. Data were presented as mean ± SD (n = 3). **P < 0.0021, ***P < 0.0002, ****P < 0.0001 in comparison to the negative control, assessed using one-factor ANOVA.

Apart from apoptosis, the involvement of autophagy in cell death mechanism represents an interesting question. This study further explored the free DTX, TQ, B-NE, and (DTX + TQ) B-NE effects on the autophagy process. As displayed in Fig. 6, the autophagic death in MCF-7 cells resulted from treatment with DTX alone significantly increased by 57.1 ± 2.0 % compared to the untreated control cells. Similarly, (DTX + TQ) B-NE increased autophagic cell death by 57.0 ± 0.8 %. However, the induction of autophagic cell death by TQ and B-NE treatments were 34.5 ± 3.1 % and 15.2 ± 0.8 %, respectively, which were not significantly changed when compared to the untreated cells. In terms of the autophagy assessment in MDA-MB-231 cells, the lone treatments with DTX, TQ, and B-NE, as well as the combination treatment with (DTX + TQ) B-NE, significantly increased the formation of autophagic vesicles by 63.1 ± 5.07 %, 85.1 ± 5.0 %, 76.3 ± 1.3 %, and 73.3 ± 7.01 %, respectively, when compared to untreated control cells (Fig. 6).

## Discussion

In this study, B-NE was successfully formulated by the sonication technique and was loaded with a combination of DTX and TQ. Blank B-NE consisted of the suspended nanodroplets with average diameters of 56.04 ± 4.00 nm with a narrow size distribution, whereas the nanodroplets of (DTX + TQ) B-NE formulation had become significantly swollen in size with 235.00 ± 10.00 nm, indicating the entrapment of the loaded drugs by dispersion within the B-NE matrix^32^. Additionally, the zeta potential values of either blank or drugs loaded B-NEs formulas were approximately below -1 mV with a low net negative charge. It has commonly been assumed that the zeta potential with a high negative value more favorable as it reflects the stability of the NE system due to the repulsion force^33^. However, many studies specified that natural or positive zeta potentials were significantly associated with the cellular uptake of the nano-delivery system, thus it would be beneficial to formulate NEs having a more positive zeta potential rather than negative for enhancing their cellular uptake during the process of delivery^34,35^. Moreover, the stability evaluations of the (DTX + TQ) B-NE exhibited small *K*s value suggesting little precipitating or floating of the suspended nanodroplets and thus could be maintained for a long time^22^.

Furthermore, the release profile of the (DTX + TQ) B-NE was noticeably higher than the free drugs combination. The poor solubility of free drugs could lead to a lower release rate resulting in lower bioavailability^6^. Therefore, the incorporation of (DTX + TQ) into the core of B-NE could improve the solubility and permeability of the loaded drugs that lead to enhancement in the drug release^24^. Moreover, the release profiles of both the DTX + TQ and (DTX + TQ) B-NE displayed a biphasic pattern with a relatively fast release of the drugs in the initial stage followed by the second stage of a slower and sustained release within 24 h, hence it would ensure the maximum efficiency of the drugs over time^7,36,37^. Similar findings have been previously reported for the release patterns of DTX from another self-nanoemulsifying drug delivery system^6^.

The cytotoxicity analysis of MCF-7 and MDA-MB-231 cells revealed that the DTX and TQ combination exhibited an antagonistic effect. However, when the combined drugs were loaded into the B-NE system, it interestingly exhibited a clear synergistic effect with a superior cytotoxicity effect. This leads to the assumption that B-NE could contribute to enhancing the DTX and TQ interaction through probably an increase in their solubility and permeability. Owing to the targeting ability of the NE system, it would facilitate the cellular uptake resulting in excessive sensitivity to the loaded drugs^24,38^. This finding is in agreement with previous studies reporting that the cytotoxicity of different combination therapy was improved when included in nano-delivery systems^7,39,40^.

Furthermore, the growth of MCF-7 and MDA-MB-231 cells were significantly suppressed when treated with the (DTX + TQ) B-NE even at lower doses of DTX with ${\text{IC}}_{50}$ value of 1.2 ± 0.97 µM and 0.5 ± 0.91 µM, respectively. However, the single DTX treatment could not cause a similar cytotoxicity rate without a higher dose when compared to the combination formula. Thereby, the cytotoxic effects of drugs were further pronounced when they were administered as a nano-formulation, which leads to a reduction in the required dose of DTX while concurrently improving its bioavailability. This finding is in agreement with numerous studies that have highlighted the remarkable effect of NEs as a delivery system in enhancing the cytotoxic effect of DTX against many other cell lines^6,41,42^.

The morphological features for MCF-7 and MDA-MB-231 cells were observed since it could contribute to the explanation of the status of cell death. Apoptosis progresses through a highly coordinated sequence of events, which are represented in specific phenotypic alterations such as DNA fragmentation and plasma membrane blebbing^43^. The MCF-7 cells treated with B-NE and (DTX + TQ) B-NE had undergone late apoptosis, as indicated by the shrinkage of cells, nuclear fragments, and the appearance of apoptotic bodies. In the case of MDA-MB-231 cells treated with (DTX + TQ) B-NE, the observation revealed noticeable cytoplasmic vacuolation that might indicate the association of the autophagic pathway.

The effect of DTX and TQ drugs and their combination in B-NE on apoptosis pathways were further assessed. It has been found that MCF-7 cells treated with B-NE alone displayed superior late apoptotic effect when compared to the other treatments. The highest early apoptotic effect in MCF-7 cells was induced by the single DTX treatment, while MDA-MB-231 cells treated with blank B-NE displayed the highest early apoptotic effect. Furthermore, despite the reduction in the DTX dose used in the combination therapy of (DTX + TQ) B-NEs when compared to the free DTX dose, both treatments remarkably exhibited an equivalent apoptotic effect in MCF-7 and MDA-MB-231cells. This result indicates that the co-incorporation of TQ and DTX into B-NE could trigger apoptotic cell death even at a lower dose of DTX. In addition, this observation further confirms the synergistic effect found with the (DTX + TQ) B-NEs formulation. In agreement with this data, Ganta et al*.*^30^ found that NEs have the potential to deliver DTX with enhanced apoptosis induction, which inhibits proliferation in ovarian cancer cells. Taken together, in MCF and MDA-MB-231 cells, the results indicate that TQ has an essential role in the combination formula since it allows a reduction in the required DTX dosage while maintaining its effectiveness through multiple pathways. When the plant derived-natural product TQ is combined with DTX and introduced by a nanoemulsion delivery system, it provides a synergistic effect in cell growth inhibition, enhancement of DNA breakage, and apoptosis induction.

Autophagy has lately gained increasing attention in cancer therapy, in a particular investigation in the highly complex interaction between apoptosis and autophagy as it remains a challenge for cancer treatment^44,45^. Autophagy appears to act as a double-edged sword that can either protect cells from apoptosis or promote apoptosis depending on many factors essentially the triggering stimuli^46,47^. In MCF-7 cells, induction of autophagy by blank B-NE was not significant when compared to the negative control, even though previous apoptosis results showed that treatment with blank B-NE remarkably increased late apoptosis. Thus, the damage promoted by blank B-NE leads to the inhibition of autophagy that seems to increase susceptibility to apoptosis. One explanation for this observation is that autophagy is restricted by the apoptotic signaling pathway due to the extensive DNA damage that would typically stimulate an apoptotic response^47^. Based on earlier findings from the morphological analysis of MCF-7 cells treated with blank B-NE, the morphology of the cells was obviously characterized with membrane blebbing, which is an ATP-dependent process that requires autophagy^47^. Therefore, in this type of cross-talking autophagy may still assist in a particular hallmark of apoptosis without leading directly to cell death. On the other hand, when the B-NE was loaded with DTX and TQ, a significant increase in the number of autophagic vesicles was detected as compared with the untreated cells in both MCF-7 and MDA-MB-231cells. These results indicate the involvement of apoptosis and autophagy activation in the (DTX + TQ) B-NE mechanism of action with a direct contribution of both processes to cell death. This finding is in line with the earlier observations derived from the apoptosis analysis, as the (DTX + TQ) B-NE showed an effective apoptotic induction. Additionally, the cytoplasmic vacuolization, observed in the morphological analysis of cell death, further emphasizes the induction of autophagy. In other words, when the combination treatment is loaded within B-NE, autophagy seems to stimulate the apoptotic pathway and contribute simultaneously and even cooperatively to lead the cell death.

## Conclusions

To the best of our knowledge, this is the first report of a novel formulation composed of DTX and TQ co-loaded into the B-NE system. This formulation aimed to minimize the effective dose of DTX in order to avoid drug toxicity. The physical characterization analysis for the developed B-NE formulation exhibited a homogenous distribution with the optimal size for drug delivery. The cytotoxicity evaluations have confirmed that the (DTX + TQ) B-NE formula has a better inhibition effect than the free combined drugs (DTX + TQ) on MCF-7 and MDA-MB-231 cells. The cell death analysis of the (DTX + TQ) B-NE indicated a possible synergistic effect of the combined drugs in stimulating autophagy and apoptosis simultaneously. The knowledge accumulated through this research highlights the potential of the B-NE as a promising candidate for the administration of the combination therapy DTX and TQ that could assist in reducing the required effective anticancer dose of the drugs for the treatment of breast cancer. Further studies are needed to define the molecular mechanisms activated by this formulation which lead to improved anticancer activity. The molecular and phenotypic heterogeneity in human breast carcinomas poses a significant limitation for designing effective treatment regimens. The highly variable sensitivity to the treatment, including plant-derived therapeutics, can be observed, and their acquired drug resistance is considered a challenge. Therefore, the clinical approach of nanoemulsion-based combination therapy consisted of various plant-derived anticancer agents administered together with the conventional chemotherapy should be superior compared to a single compound in cancer treatment, which can be attributed to its potential to affect multiple signaling pathways in cancer cells that may delay the development of drug resistance. In view of that, further research is needed to identify new nano-based combination therapies that include plant-derived compounds with well-validated anticancer properties to further improve the treatment options within clinical oncology.
